# Review of Diagnostic Tests for Detection of *Mycobacterium bovis* Infection in South African Wildlife

**DOI:** 10.3389/fvets.2021.588697

**Published:** 2021-01-28

**Authors:** Netanya Bernitz, Tanya J. Kerr, Wynand J. Goosen, Josephine Chileshe, Roxanne L. Higgitt, Eduard O. Roos, Christina Meiring, Rachiel Gumbo, Candice de Waal, Charlene Clarke, Katrin Smith, Samantha Goldswain, Taschnica T. Sylvester, Léanie Kleynhans, Anzaan Dippenaar, Peter E. Buss, David V. Cooper, Konstantin P. Lyashchenko, Robin M. Warren, Paul D. van Helden, Sven D. C. Parsons, Michele A. Miller

**Affiliations:** ^1^Division of Molecular Biology and Human Genetics, Faculty of Medicine and Health Sciences, DSI-NRF Centre of Excellence for Biomedical Tuberculosis Research, South African Medical Research Council Centre for Tuberculosis Research, Stellenbosch University, Cape Town, South Africa; ^2^Veterinary Wildlife Services, South African National Parks, Kruger National Park, Skukuza, South Africa; ^3^Ezemvelo KwaZulu-Natal Wildlife, Mtubatuba, South Africa; ^4^Chembio Diagnostic Systems, Medford, NY, United States

**Keywords:** bovine tuberculosis, diagnostics, *Mycobacterium bovis*, South African wildlife, immunological assays, direct detection of mycobacteria, cytokine release assays, gene expression assays

## Abstract

Wildlife tuberculosis is a major economic and conservation concern globally. Bovine tuberculosis (bTB), caused by *Mycobacterium bovis* (*M. bovis*), is the most common form of wildlife tuberculosis. In South Africa, to date, *M. bovis* infection has been detected in 24 mammalian wildlife species. The identification of *M. bovis* infection in wildlife species is essential to limit the spread and to control the disease in these populations, sympatric wildlife species and neighboring livestock. The detection of *M. bovis*-infected individuals is challenging as only severely diseased animals show clinical disease manifestations and diagnostic tools to identify infection are limited. The emergence of novel reagents and technologies to identify *M. bovis* infection in wildlife species are instrumental in improving the diagnosis and control of bTB. This review provides an update on the diagnostic tools to detect *M. bovis* infection in South African wildlife but may be a useful guide for other wildlife species.

## Introduction

*Mycobacterium bovis* is a member of the *Mycobacterium tuberculosis* complex (MTBC), a group of genetically related mycobacterium species that cause tuberculosis in a range of mammals (1). Of all MTBC members, *M. bovis* has the widest host range and causes bovine tuberculosis (bTB) in domestic animals, livestock, wildlife, and humans (2). Globally, the eradication of *M. bovis* is hampered by the existence of wildlife reservoirs that serve as recurrent sources of infection, posing a threat for spillover to livestock at the livestock-wildlife interface, and other sympatric wildlife species (3).

In South Africa, *M. bovis* is the most common cause of wildlife tuberculosis, with two of the largest wildlife reserves, the Kruger National Park (KNP) and Hluhluwe-iMfolozi Park (HiP), declared endemic for *M. bovis* (2, 4). The KNP and HiP are adjacent to communal lands where livestock graze freely (5, 6), risking the spillover of bTB from wildlife to domestic livestock. This may have regulatory consequences and subsequent imposed trade restrictions (6, 7). In addition, the detection of bTB in wildlife can lead to quarantine of wildlife premises and threaten conservation and tourism, which can have environmental and socio-economic implications (8).

The development and optimisation of diagnostic tools to identify *M. bovis* infection is a crucial step to identify affected individuals to manage transmission. However, the validation of diagnostic tests for wildlife bTB is limited by access to large numbers of high-quality samples from confirmed infected and uninfected species (9–11). Due to logistical challenges, it can be difficult to confirm *M. bovis* infection, especially from suspected cases using antemortem samples.

**Figure 1:**
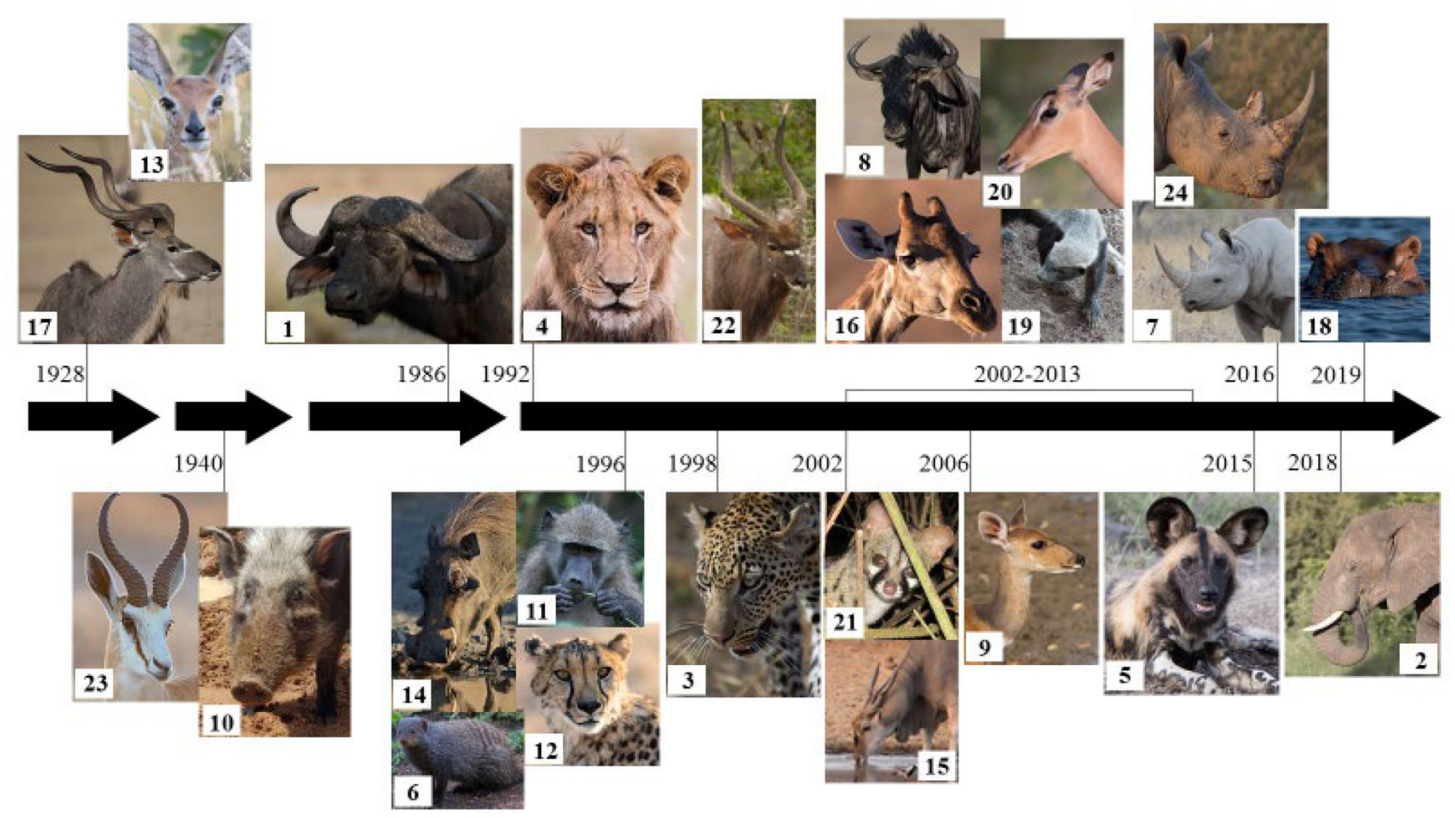
Index cases of *Mycobacterium bovis* infection in free-ranging wildlife species in South Africa over the last century (35). Species correspond to the superscript numbers in Table 1.

With the advent of new techniques and tools to detect *M. bovis* infection in wildlife, our understanding of bTB continues to evolve. To date, *M. bovis* infection has been detected in 24 mammalian wildlife species in South Africa (Table 1) with most cases in these species identified within the last decade (Figure 1). The development of diagnostic tools to identify infected hosts is essential to limit the spread of bTB and to control the disease. The aim of this review is to provide an update on recent diagnostic developments for wildlife bTB in South Africa.

**Table 1:** Free-ranging wildlife species confirmed to be infected by *Mycobacterium bovis* in South Africa, based on location, along with references.

**Common name**	**Species**	**KNP**	**GKNP**	**HiP**	**KZN**	**MGR**	**SNR**	**Other**	**Reference/s**
African buffalo	*Syncerus caffer*^1^	√	√	√	√	√	√	Mpumalanga	(4, 12–14)
African elephant	*Loxodonta africana*^2^	√	–	–	–	–	–	–	(15)
African leopard	*Panthera pardus*^3^	√	√	√	–	–	√	–	(16, 17)
African lion	*Panthera leo*^4^	√	–	√	√	√	√	–	(14, 16)
African wild dog	*Lycaon pictus*^5^	√	–	√	–	–	–	–	(18, 19)
Banded mongoose	*Mungos mungo^6^*	√	–	–	–	–	–	–	(20)
Black rhinoceros	*Diceros bicornis*^7^	√	–	–	–	–	–	–	(21)
Blue wildebeest	*Connochaetes taurinus*^8^	–	√	–	–	–	–	–	(6)
Bushbuck	*Tragelaphus scriptus*^9^	√	–	–	–	–	–	–	(22)
Bush pig	*Potamochoerus porcus*^10^	–	–	√	–	–	–	–	(12, 16)
Chacma baboon	*Papio ursinus*^11^	√	–	√	√	–	–	Limpopo	(4, 16, 23–25)
Cheetah	*Acinonyx jubatus*^12^	√	√	–	–	–	–	Limpopo	(16, 23, 26)
Common duiker	*Syvicapra grimmia*^13^	–	–	–	–	–	–	Eastern Cape	(27)
Common warthog	*Phacochoerus africanus*^14^	√	√	–	√	–	–	–	(28, 29)
Eland	*Taurotragus oryx*^15^	–	√	√	–	–	–	–	(2, 16)
Giraffe	*Giraffa camelopardalis*^16^	–	√	–	–	–	–	–	(30)
Greater kudu	*Tragelaphus strepsiceros*^17^	√	√	√	√	–	√	–	(4, 27, 31)
Hippopotamus	*Hippopotamus amphibius*^18^	–	√	–	–	–	–	–	(32)
Honey badger	*Mellivora capensis*^19^	√	–	√	–	–	–	–	(16)
Impala	*Aepyceros melampus*^20^	√	–	–	–	–	–	–	(16)
Spotted genet	*Genetta tigrina*^21^	√	–	√	–	–	–	–	(16, 17)
Nyala	*Tragelaphus angasii*^21^	–	–	–	–	–	–	Gauteng	(4)
Springbok	*Antidorcas marsupialis*^23^	–	–	–	–	–	–	–	(33)
White rhinoceros	*Ceratotherium simum*^24^	√	–	–	–	–	–	–	(34)

*Superscript numbers correspond to species in Figure 1*.

*KNP, Kruger National Park; GKNP, Greater Kruger National Park; HiP, Hluhluwe-iMfolozi Park; KZN, KwaZulu-Natal; MGR, Madikwe Game Reserve; SNR, Spioenkop Nature Reserve*.

## Direct Detection

### Microscopy and Acid-Fast Stain

Direct staining of sample material with Ziehl-Neelsen stain can provide presumptive diagnosis of mycobacterial infection. Impression smears from lesions in tissues or secretions can provide a rapid screening technique in cases in which bTB is suspect (21). Staining of tissues is also useful for postmortem and antemortem diagnosis in limited cases in which biopsy or other relevant samples such as lymph node may be available (36). Although the presence of acid-fast bacteria provides a presumptive diagnosis, paucibacillary infection may result in a false-negative result using this method (11, 37). In addition, non-tuberculous mycobacteria and non-mycobacterial organisms, such as *Nocardia*, can stain positive, with a false positive result (38). Therefore, methods that provide differentiation from NTM and speciation within the *Mycobacterium tuberculosis* complex are important for a definitive diagnosis (39, 40).

### Mycobacterial Culture

Mycobacterial culture is the gold standard of *M. bovis* infection with speciation confirmed by polymerase chain reaction (PCR). In recent years, there have been advances in techniques to improve direct detection of *M. bovis* infection in wildlife; however, most procedures still require growing the organism to measurable levels using different mycobacterial culture methods. Due to the inherent slow growth of mycobacteria, the development of improved culture techniques using different media has been investigated.

*Mycobacterium bovis* has been isolated from both livestock and wildlife samples using solid and liquid culture media. In a study by the National Veterinary Services Laboratory (USA), the liquid BACTEC 12B media supported significantly higher detection of *M. bovis* compared to the liquid MGIT 960 (93.1 vs. 81.9%, respectively) (both Becton Dickinson, Franklin Lakes, NJ, USA), and both outperformed solid Lowenstein-Jensen and Middlebrook media (41). However, the authors concluded that MGIT 960 media was favored due to increased specific recovery of MTBC and decreased time to positivity.

The BACTEC™ MGIT™ is an automated mycobacterial growth detection system used in routine human tuberculosis diagnostic settings, that has been used to culture MTBC organisms from postmortem tissue samples as well as antemortem bronchoalveolar lavage, elephant trunk wash fluid, and oropharyngeal swab samples from wildlife (42–44). Application of these techniques has permitted antemortem diagnosis of *M. bovis* infection in African lion (*Panthera leo*), African wild dog (*Lycaon pictus*) and white rhinoceros (*Ceratotherium simum*) (36, 42, 45).

More recently, TiKa-MGIT (TiKa Diagnostics, UK), a novel specialized culture medium (containing a mild decontamination step and growth enhancer steps), with the unique ability to stimulate MTBC growth when used together with the BACTEC™ MGIT™ system, has been used to improve sensitivity of mycobacterial culture, even from samples with low mycobacterial numbers (46). Greater use of this technique may enhance detection of MTBC infection in wildlife in which paucibacillary samples or those heavily contaminated with other environmental bacteria have resulted in false-negative culture results using conventional methods.

### PCR

Various PCR-based methods have been developed and adapted to identify specific MTBC organisms, based on detecting the presence of mycobacterial DNA, from either cultured isolates or directly from ante or postmortem samples (43, 47). Two emerging PCR-based molecular tools for wildlife samples include VetMAX™ *M. tuberculosis* complex PCR kit (Thermo Fischer Scientific, Waltham, MA, USA) and cartridge-based GeneXpert® (Cepheid, Sunnyvale, CA, USA) technology.

The VetMAX™ MTBC PCR kit was developed for rapid detection of *M. bovis* in cattle lymph nodes and other tissues. The real-time PCR targets the IS*6110* insertion element found in MTBC, and therefore, should be applicable to samples from any animal species. Use with samples from South African wildlife has been described with successful detection of MTBC DNA in lymph node tissue homogenates and respiratory secretions from buffaloes, white rhinoceros, and African elephants (47). Although useful for diagnosis of MTBC infection in species with limited host-based diagnostic tests, the performance of the assay requires DNA extraction of samples and a facility that can perform PCR, which restricts its use to diagnostic or research laboratories.

The GeneXpert® is a cartridge-based automated screening system endorsed by the World Health Organization (48). Two assays, Xpert® MTB/Rif and Xpert® MTB/Rif Ultra have been developed for the detection of *Mycobacterium tuberculosis* infection and resistance to rifampicin in humans via PCR (43). This technology allows the detection of MTBC deoxyribonucleic acid (DNA) using a simplified and standardized method and is effective in high throughput diagnostic or research settings. In addition, it is often available in regions where the human tuberculosis burden is high and in regions where laboratories for mycobacterial culture may not exist.

This rapid, simple-to-use, automated platform is currently being adapted and optimized to detect *M. bovis* from wildlife samples (49) including postmortem tissue homogenates and antemortem bronchoalveolar lavage samples, trunk wash fluids, and oral swabs from elephants, rhinoceroses, and African buffaloes (47). The Xpert® MTB/RIF Ultra assay has also been used to confirm the presence of MTBC DNA directly from tissue homogenates from cheetah (*Acinonyx jubatus*) (26).

The implementation of GeneXpert® has already played a major role in implementing centralized human tuberculosis diagnosis, increased the number of detected cases, and shortened the diagnostic delay, despite the limitation that it only detects MTBC DNA rather than viable bacilli (48). Therefore, this benchtop system, which can be used with a range of sample types, may provide an alternative or ancillary diagnostic technique for tuberculosis detection in South African wildlife as well as other populations globally, especially when mycobacterial culture is unavailable.

## Speciation and Strain Identification

The most common molecular typing tools used to genetically differentiate *M. bovis* are: (i) region of difference (RD); (ii) spacer oligonucleotide typing (spoligotyping); (iii) variable number of tandem repeats (VNTR) typing of mycobacterial interspersed repetitive units (MIRU); and (iv) next generation sequencing (50–53).

### Region of Difference Analysis

Region of difference analysis is used to speciate MTBC members and allows rapid identification of *M. bovis* in culture isolates (54). In wildlife, this technique has resulted in differentiation of animal adapted MTBC strains, such as *M. mungi* in mongooses, *M. suricate* in meerkats, and *M. orygis* in a buffalo (55–57). This speciation is important for understanding the epidemiology of infection in different animal hosts.

### Spoligotyping

Spoligotyping is commonly used to detect and identify the genotype of MTBC isolates to determine the phylogenetic relationships between organisms from specific geographical regions and to track sources of infection (Table 2). Previously, data suggested that a single dominant strain of *M. bovis* circulated within wildlife in specific geographical locations in South Africa. Two geographically distinct spoligotype patterns existed in South African wildlife and were believed to have been caused by different progenitor strains (12, 16). A ‘Kruger’ spoligotype (SB*0121*) and a ‘KwaZulu Natal’ spoligotype (SB*0140*) were isolated in wildlife in Greater Kruger National Park (GKNP) and KwaZulu Natal, respectively (12). In a recent study, Sichewo et al. [2020] analyzed samples from cattle and wildlife in KwaZulu Natal and found that although SB*0130* was the dominant pattern, these *M. bovis* isolates were comprised of 29 different strains based on VNTR (58). These results suggest a high level of genetic diversity in this area. These results also support the hypothesis that there are several different *M. bovis* strains circulating in South Africa. Since spoligotypes SB*0120* and SB*0121* are the predominant patterns circulating in animals worldwide, as well as finding SB*0140* in Asia, Europe and America, it is highly likely that there were multiple introductions with movement of livestock between countries, and inter-species transmission at livestock-wildlife interfaces in South Africa (59). The genetic diversity of *M. bovis* in South Africa may also be indicative of genetic divergence, ongoing transmission, as well as movement of infected animals (4), but further epidemiological studies are required.

**Table 2:** Spoligotype (SB) numbers of *Mycobacterium bovis* isolates identified in free-ranging wildlife in South Africa, based on location of the infected species, with references.

**SB #**	**KNP**	**GKNP**	**HiP**	**KZN**	**MGR**	**Reference/s**
SB*0121*	√	√	√	√	–	(12, 28, 30, 34, 53, 58)
SB*0130*	–	√	√	√	√	(6, 12, 16, 53, 58)
SB*0140*	–	–	√	√	√	(4, 28, 53)
SB*1474*	–	–	√	√	–	(12)
SB*0120*	–	√	–	–	–	(6, 28)
SB*1275*	–	√	–	–		(28)
SB*1388*	–	√	–	–	–	(28)
SB*0294*	–	√	–	–	–	(30)
SB*2200*	–	√	–	–	–	(6)

*GKNP, Greater Kruger National Park (adjacent private reserves including Marloth Park); KNP, Kruger National Park; HiP, Hluhluwe iMfolozi Park; KZN, KwaZulu-Natal; MGR, Madikwe Game Reserve*.

### VNTR Typing

Variable number tandem repeat (VNTR) typing of *M. bovis* is also used to evaluate transmission by assessing the genetic homology of different isolates. For example, the same VNTR genotype (and spoligotype) of *M. bovis* was found in cattle and wildlife species in the Greater Kruger National Park Complex, providing evidence that spillover had occurred (7). Studies have shown that VNTR typing is more discriminatory than spoligotyping and often used in combination. These techniques were used to characterize *M. bovis* isolates from wildlife in Hluhluwe-iMfolozi Park, which is endemic for *M. bovis* (12). Using VNTR and spoligotyping, the patterns suggested that multiple introductions had occurred and provided a basis for evaluating the epidemiology of bTB in the park. A larger study used both techniques to describe the high genetic diversity of *M. bovis* strains circulating in livestock and wildlife populations around South Africa and provided data that showed inter-species transmission especially at wildlife/livestock interfaces (6, 58). Molecular characterization of *M. bovis* isolates is crucial for epidemiological investigation of outbreaks, especially involving multiple species.

### NGS

Next generation sequencing (NGS) of MTBC has increased resolution and discriminatory power compared to other molecular-based genotyping methods. The generation of whole genome sequences (WGS) allows distinct genetic profiles to be compared at the nucleotide level, and MTBC molecular epidemiology and genetic diversity can be investigated with greater resolution (53). In common warthogs (*Phacochoerus africanus*), NGS permitted the differentiation of MTBC isolates revealing two distinct clades of *M. bovis* that were not differentiated using spoligotyping (28); therefore, NGS could improve molecular epidemiological investigations of MTBC infections by resolving transmission events or shedding light on genetic divergence.

A study that analyzed WGS data of 17 *M. bovis* isolates from different host species in different locations within South Africa revealed distinct regional genomic characteristics (53). Different host species in the KNP clustered together suggesting the introduction of a single progenitor *M. bovis* strain which led to clonal expansion (53).

High quality WGS is currently dependent on mycobacterial culture due to the requirement for large quantities of DNA for WGS library preparation. Furthermore, a key limitation of WGS is that unless large sample sizes are used, directionality of transmission between species cannot be determined (4, 60–62). The technology for producing diagnostic-quality MTBC WGS directly from specimens is being developed, although still in its infancy. The greater resolution offered by NGS, in combination with a growing availability of *M. bovis* isolates with WGS, will provide a database for future epidemiological investigations in wildlife, similar to what is being done to trace livestock tuberculosis outbreaks (63).

## Indirect Detection

Despite improvements in directly identifying MTBC organisms, which provides a definitive diagnosis by confirming the presence of the pathogen in a sample or animal, detection of host immune responses remains the principal method used to diagnose *M. bovis* infection in wildlife. Indirect methods rely on measuring the anamnestic cell-mediated or humoral immune responses to *M. bovis* antigens in different species (Table 3).

**Table 3:** Indirect diagnostic tools to detect immune sensitization to *Mycobacterium bovis* in free-ranging wildlife species in South Africa.

**Species**	**SCITT**	**IGRA**	**CRA**	**Serology**	**GEA**	**References**
*Acinonyx jubatus*	√	–	–	√	√	(26)
*Ceratotherium simum*	–	√	–	–	–	(64)
*Crocuta crocuta*	–	–	–	–	√	(65)
*Diceros bicornis*	–	√	–	√	–	(66, 67)
*Lycaon pictus*	√	√	–	√	–	(18, 19)
*Panthera leo*	√	–	–	√	√	(68–72)
*Phacochoerus africanus*	√	–	√	√	√	(28, 29, 73–75)
*Syncerus caffer*	√	√	√	√	√	(13, 76–83)

*SCITT, single comparative intradermal tuberculin test; IGRA, interferon gamma release assay; CRA, cytokine release assay; GEA, gene expression assay*.

Assays that detect humoral responses use samples (sera) that are typically more readily available than assays based on cell-mediated immunity (CMI), which often require timely processing of fresh whole blood. However, CMI-based assays have shown greater sensitivity in many species (10, 68) and may detect mycobacterial infections earlier than serological assays (84). This may vary with species as more recent studies have shown that humoral responses are better indicators of MTBC infection in some species such as common warthogs and African elephants (29, 85).

The MTBC antigens used to elicit *in vitro* immune responses in wildlife can either be complex antigens, such as *M. bovis* purified protein derivative (PPD) or specific mycobacterial peptides (culture filtrate protein 10 and early secretory antigenic target 6 protein) like those in the QuantiFERON® TB-Gold Plus (QFT) system (Qiagen, Venlo, Limburg, Netherlands). The QFT system is a set of tubes, with standardized antigenic peptides and controls, that has been used with whole blood to stimulate MTBC-specific cytokine production *in vitro* from a number of wildlife species including African buffaloes, common warthogs, African lions, African wild dogs, white rhinoceros and cheetahs (9, 43, 65, 69, 73, 76, 86). These samples can be processed in most veterinary clinics, avoiding the need to get samples to a laboratory within the required 8–10 h for subsequent cytokine release assays. However, species-specific cytokine detection methods, such as enzyme-linked immunosorbent assays (ELISA) or gene expression assays (GEA), still need to be developed and validated for many wildlife species. In addition, cytokine biomarkers for tuberculosis often vary between species; for example, interferon-gamma (IFN-γ) release assays (IGRA) are available for African wild dogs, white rhinoceroses, and African buffaloes, but *CXCL9* and *CXCL10* gene expression assays are used as diagnostic assays for African lion, cheetah, and common warthog (43, 65, 69, 73).

*In vivo* CMI tests and the presentation of clinical disease may also be used as indirect methods of detecting bTB. However, clinical assessment is an insensitive tool for diagnosis as clinical disease is typically only observed in advanced stages and may only be practical for use in captive wildlife (68, 87).

### In vivo

#### Single Intradermal Comparative Tuberculin Test

The single comparative intradermal tuberculin test (SCITT) relies on stimulating a CMI response *in vivo* with the intradermal injection of bovine and avian PPDs, after which a memory response is measured by a delayed-type hypersensitivity reaction. The SCITT is applied across multiple wildlife species including African buffalo, African wild dog, African lion, cheetah, leopard, antelope, and common warthog, however, validation has only been performed in a few of these species (70, 74, 77).

The need to calculate a SCITT species-specific cut-off value with appropriate study cohorts was highlighted in a study in which previously published criteria used to interpret SCITT for lions led to false positive results in 54% of animals tested (70, 71). The study by Roos et al. (74) in a bTB endemic cohort of common warthogs demonstrated that *M. bovis*-infected animals develop a measurable delayed-type hypersensitivity reaction to *M. bovis* PPD and the sensitivity and specificity of the SCITT, using common warthog-specific cut-off values, were 81 and 100%, respectively (74). Currently, the SCITT is performed in African buffaloes using the interpretation criteria for cattle, leading to suboptimal sensitivity (78). In some species, such as elephants and rhinoceroses, the SCITT is unreliable and not recommended for use, although the response to intradermal injection of specific antigens instead of PPDs should be investigated as a potential option (88). Due to the cost and logistical problems associated with performing the SCITT in wild animals, blood-based assays, namely cytokine release assays or GEA, that require only a single immobilization and blood collection and can deliver a result in <72 h, would be valuable wildlife tools for bTB surveillance and diagnosis.

### In vitro

#### Cytokine Release Assays

The cytokine IFN-γ is the most used biomarker to quantify *in vitro* CMI responses to MTBC in humans, livestock, and wildlife. Commercially available ELISAs have been evaluated and optimized to detect cytokine production in specific wildlife species; the Bovigam® and Bovigam® 2G ELISAs (Prionics AG, Schlieren-Zurich, Switzerland), ruminant *cattletype*® IFN-gamma ELISA (INDICAL, Inc., San Francisco CA, USA), and Mabtech bovine interferon-gamma (IFN-γ) ELISA^PRO^ kit (Mabtech, Nacka Strand, Sweden) detect African buffalo IFN-γ (76, 77, 79), the Bovigam® detects nyala IFN-γ (89), the Quantikine® canine IFN-γ ELISA (R&D Systems, Inc., Minneapolis, MN, USA) detects wild dog IFN-γ (45), and the equine IFN-γ ELISA^PRO^ (Mabtech) detects white rhinoceros IFN-γ (9). Moreover, alternative biomarkers to IFN-γ have been investigated, with the chemokine IFN-γ-inducible protein-10 (IP-10) shown to be a biomarker of immune activation in African buffaloes and common warthogs (13, 75).

Since no single cytokine release assay has perfect sensitivity and specificity (80), biomarker panels are being investigated to improve diagnostic performance. The parallel measurement of IFN-γ and IP-10 has been shown to maximize the detection of *M. bovis*-infected African buffaloes while maintaining specificity (13). In some wildlife species, it may be difficult to develop cytokine release assays due to the lack of species-specific reagents; in these cases, GEA may be an alternative method of determining antigen-specific immune responses.

#### Gene Expression Assays

Gene expression analysis quantifies a change in antigen-specific cytokine gene transcription reflecting an immune response. These assays have shown utility in the diagnosis of *M. bovis* infection in African lions and cheetahs by measuring the expression of *CXCL9* mRNA (26, 69), in African buffaloes by measuring the expression of IFN-γ mRNA (81) as well as in warthogs (73) and white rhinoceros (90) by measuring the expression of *CXCL10* mRNA. Furthermore, GEA has been used to detect immune sensitization to *M. bovis* in spotted hyena (*Crocuta crocuta*), by measuring the expression of *CXCL9* and *CXCL11* mRNA (65). In species such as African elephants, this approach could be used to circumvent the requirement to produce unique species-specific reagents, such as antibodies to elephant cytokines. Published cytokine gene primer sequences may facilitate use of these assays by additional laboratories without the requirement to share non-commercially available reagents.

#### Serological Assays

Antigen-specific circulating antibodies can be used to differentiate between *M. bovis-*infected and -uninfected individuals in some species. In common warthogs, an indirect in-house PPD ELISA and the TB ELISA-VK® kit (Vacunek, Bizkaia, Spain) were able to detect *M. bovis*-specific antibodies with high sensitivity and specificity (29). However, variable test performance was observed when a commercial TB ELISA (IDEXX Laboratories Inc., Westbrook, ME, USA) was used to detect *M. bovis*-specific antibodies in naturally infected African buffaloes from herds with different bTB prevalence (82). The low sensitivity of serological assays for buffaloes was also observed when serum antibodies were detected to specific mycobacterial peptides using rapid lateral flow assays (91), which suggests that these may not be useful diagnostic tests for this species. In contrast, the Dual Path Platform Vet TB Assay (DPP® VetTB) for Cervids® (Chembio Diagnostic Systems, Medford, NY, USA) has been shown to be a sensitive test to diagnose *M. bovis* infection in common warthogs (29) and the Elephant TB STAT-PAK® Assay (Chembio Diagnostic Systems) and the DPP® VetTB for Elephants (Chembio Diagnostic Systems) have been used to determine the seroprevalence of MTBC infection in African elephants in KNP (85). The Elephant TB STAT-PAK® and DPP VetTB® assays have also been shown to distinguish between *M. bovis*-infected and -unexposed African lion populations (68, 69, 72). These species-non-specific rapid tests have also been useful for identifying *M. bovis*-infected white and black rhinoceros, and a cheetah (26, 34, 66). Sensitivity of serological tests varies between species, although providing a useful tool for retrospective surveys (68, 85).

## Discussion

Diagnostic tests are used by veterinary clinicians, researchers, and animal health regulatory staff to detect infection with *M. bovis* in individual animals as well as screen herds or perform surveillance in populations. A recent review described current antemortem and postmortem diagnostic tests for wildlife from a global perspective (92). Therefore, the current review focused on the use of diagnostic test specifically for South African wildlife species since application of techniques can vary between hosts as well as geographical location (10, 93). In addition, to accurately interpret results, a diagnostic test should be validated and “fit for purpose,” according to the OIE (11). For example, the OIE has validated the tuberculin skin test in cattle to permit international trade (11) and in South Africa, this has also been approved for certifying African buffaloes for translocation. Although many routine tests for bTB (developed in domestic animals) are used in wildlife, most have not been validated for these species (11).

In general, tests which rely on direct detection of *M. bovis* are less likely to vary significantly in test performance between species and those validated in a domestic species should be relevant for other related species. However, it is important to be aware of variation in the amount and distribution of the pathogen in different hosts (87). Histological examination may reveal differences in specific pathological changes between species, but the presence of a granuloma, especially with acid-fast positive bacteria present, is a presumptive diagnosis for bTB (10, 11, 94). In addition, collection of respiratory secretions by nasal swabs/lavage, oropharyngeal swabs, and bronchoalveolar lavage for mycobacterial culture or mycobacterial PCR have been applied across a wide range of wildlife species, with positive results confirming infection (36, 42, 43). However, the location of the infection, sporadic shedding, suboptimal sensitivity of mycobacterial culture, and inability of PCR to distinguish between live and dead bacteria are limitations to direct detection techniques for bTB diagnosis, especially antemortem. According to OIE guidelines, “detection of infection in a wildlife population requires bacteriological investigation or the use of a valid testing method for the species involved (the tuberculin test is not effective in all species) together with epidemiological analysis of information” (11).

In contrast to direct detection methods, indirect detection relies on the host's immune responses which can vary significantly between species. This requires knowledge of the immunology of each host species, which is often limited. In addition, understanding of pathogenesis and changes in associated immunological responses are important in appropriate application of indirect tests. One of the major limitations of these assays is that they often require species-specific reagents, which may not be available, and are costly and time-consuming to develop. Acknowledging that validation of tests for use in wildlife may be challenging due to access to adequate numbers of samples from known infected and uninfected individuals, the OIE has provided a validation pathway for provisional recognition of tests that can be used in specific applications (11). Modification of pre-existing tests that have been validated for a domestic species may be provisionally validated in a related wildlife species using a minimum of 10 positive and 10 negative well-characterized reference samples, while for a new test, the numbers increase to 30 each (11). The provisional validation of tests such as IGRAs for white rhinoceros, African buffaloes, and wild dogs demonstrates that this can be accomplished (64, 79, 95). The OIE criteria make it feasible to provide sufficient information for animal health authorities to approve the use of the test in a specified host species for a specific purpose (such as testing animals being moved or surveillance). Therefore, this review of indirect, blood-based assays that can accurately detect *M. bovis* infection in specific species demonstrates the potential these tests have for being approved tests for wildlife in the future.

Diagnostic tests for wildlife in other countries are like those described for South African wildlife (10, 93, 96, 97). Direct detection techniques for wildlife TB do not vary significantly between countries. There may be some species in which *M. bovis* is shed in non-respiratory secretions or infection is localized in specific organs, however the methods used to detect bacilli (i.e., microscopy, culture, PCR) are applicable to different sample types (3, 87, 97). In contrast, immunological assays can vary between species, and may be more limited due to lack of species-specific or cross-reactive reagents. The tuberculin skin test is commonly used in a wide range of wildlife species but rarely validated (10, 96, 97). Non-specific or cross-reactions may be more common in some species such as tapirs, camelids, and orangutans, which leads to recommendations against using the test in some groups of animals (97). Interferon gamma release assays have been used in badgers, cervids, bovids, and primates with variable sensitivity and specificity (10, 93, 96, 97). Serological assays, such as STAT-PAK, DPP, and multi-antigen print immunoassay (MAPIA), have also been used in a range of species, however, the humoral response is often only detected later in disease development although elephants, camelids, and suids appear to develop robust early responses (97–100). Therefore, the clinician selecting tests for diagnosing TB in wildlife should base the choice on knowledge of species-specific immune responses and pathogenesis. With the criteria provided by the OIE, more assays will potentially be provisionally approved which will facilitate TB screening of wildlife for movement and surveillance.

## Summary and Conclusion

During the last decade, an increasing number of wildlife species have been confirmed to be infected with *M. bovis* in South Africa (Figure 1). The identification of additional host species infected with *M. bovis* may be due to an increase in transmission or an improvement in detection, the latter being at the forefront of research. Direct detection of *M. bovis* remains difficult and time-consuming due to the paucity of bacilli in most antemortem samples, together with the slow growth of mycobacteria. However, development of rapid PCR assays may improve direct detection of MTBC in the future, particularly since some of the technology can be performed in a field setting.

Indirect detection of *M. bovis* through quantification of immune responses elicited by infection, especially *in vitro* tests, has key advantages in wild animals, and remains the cornerstone of wildlife bTB testing. The development of species-specific reagents and the identification of cross-reactive reagents, as well as standardized kits, will further improve the detection of *M. bovis* in wildlife species.

## Author Contributions

All authors listed have made a substantial, direct and intellectual contribution to the work, and approved it for publication.

## Conflict of Interest

The authors declare that the research was conducted in the absence of any commercial or financial relationships that could be construed as a potential conflict of interest.
